# COVID-19 Models for Hospital Surge Capacity Planning: A Systematic Review

**DOI:** 10.1017/dmp.2020.332

**Published:** 2020-09-10

**Authors:** Michael G. Klein, Carolynn J. Cheng, Evonne Lii, Keying Mao, Hamza Mesbahi, Tianjie Zhu, John A. Muckstadt, Nathaniel Hupert

**Affiliations:** Department of Marketing and Business Analytics, San Jose State University, San Jose, CA; School of Operations Research and Information Engineering, and Cornell Institute for Disease and Disaster Preparedness, Cornell University, Ithaca, NY; Departments of Population Health Sciences and of Medicine, Weill Cornell Medicine, and Cornell Institute for Disease and Disaster Preparedness, Cornell University, New York, NY

**Keywords:** coronavirus, COVID-19, hospital, pandemic, surge capacity

## Abstract

**Objective:**

Health system preparedness for coronavirus disease (COVID-19) includes projecting the number and timing of cases requiring various types of treatment. Several tools were developed to assist in this planning process. This review highlights models that project both caseload and hospital capacity requirements over time.

**Methods:**

We systematically reviewed the medical and engineering literature according to Preferred Reporting Items for Systematic Reviews and Meta-Analyses (PRISMA) guidelines. We completed searches using PubMed, EMBASE, ISI Web of Science, Google Scholar, and the Google search engine.

**Results:**

The search strategy identified 690 articles. For a detailed review, we selected 6 models that met our predefined criteria. Half of the models did not include age-stratified parameters, and only 1 included the option to represent a second wave. Hospital patient flow was simplified in all models; however, some considered more complex patient pathways. One model included fatality ratios with length of stay (LOS) adjustments for survivors versus those who die, and accommodated different LOS for critical care patients with or without a ventilator.

**Conclusion:**

The results of our study provide information to physicians, hospital administrators, emergency response personnel, and governmental agencies on available models for preparing scenario-based plans for responding to the COVID-19 or similar type of outbreak.

Commonly known as the *novel coronavirus*, severe acute respiratory syndrome coronavirus 2 (SARS-CoV-2) caused the 2019 infectious disease called *coronavirus disease* (COVID-19). On January 30, 2020, the World Health Organization (WHO) declared the COVID-19 outbreak a Public Health Emergency of International Concern (PHEIC).^1^ As cases spread around the world, the WHO classified COVID-19 as a pandemic on March 11, 2020.^2^ As of July 11, 2020, more than 12.5 million COVID-19 cases occurred in 188 countries, resulting in over 560 000 deaths. In the United States alone, over 3.2 million confirmed cases led to over 134 000 lives lost due to COVID-19.^3^

The COVID-19 pandemic caused unprecedented stress on health care systems around the globe, requiring treatment capabilities and resources exceeding “normal” emergency surge capacity. Radical efforts to increase treatment space were undertaken, ranging from state-wide cancellation of elective surgeries to exhortations for hospitals to double medical and surgical ward beds (eg, in New York State). At New York–Presbyterian’s Weill Cornell Medical Center, hospital administrators canceled elective procedures, then converted operating rooms and post-anesthesia care units to intensive care units (ICUs). This effort created a 50% increase in ICU capacity.^4^ However, the pandemic severely taxed New York hospitals in late March and April 2020. Personal protective equipment (PPE) was scarce, isolation capacity was insufficient, critical resource supply chains were strained, emergency departments were overwhelmed, and unstable patients were transferred between hospitals.^5^

In a large medical center in New York City (NYC), 23.6% of the first 1000 COVID-19 patients were admitted or transferred to an ICU. COVID-19 patients in these ICUs required very long length of stays (LOS) with a median of 23 days. Furthermore, the challenges extended beyond bed capacity; 57.6% of patients admitted to ICU needed a ventilator and 35.2% needed dialysis.^6^ Naturally, concerns were reported that deaths due to shortages of ventilators and dialysis machines could have been avoided if hospitals had enough critical equipment and personnel to meet the needs of COVID-19 patients.^5,7,8^

Planning for resources needed to respond to the COVID-19 virus or future pandemic is based on projecting the number and timing of cases requiring various types of treatment. Several tools were developed to assist hospital administrators, physicians, emergency response personnel, and governmental agencies in this planning process. These tools were typically used to consider a variety of possible scenarios at the beginning of the pandemic. In places where the peak of the first wave had already occurred, hospital surge capacity planning tools can help prepare for future waves. Currently, in the United States, a surge of new COVID-19 cases is occurring in multiple states that have not yet experienced a major first wave but nevertheless have relaxed physical distancing measures. Resumption of large group gatherings and the occurrence of mass protests in many parts of the country may be contributing to the current rise of cases.^9^

This study highlights planning models that can be used to estimate hospital capacity requirements due to surges of patients with COVID-19. Typically, for a planning horizon of 1 month or longer, the models can be used to consider different scenarios with different parameters. For each user-defined scenario, these tools identify an epidemic curve of the expected number of COVID-19 cases per day and the expected hospital occupancy per day in medical-surgical wards and ICUs.

We provide the input parameters, highlight key features, and explain the output that can be produced from each model. We compare distinguishing features and provide a discussion on the usefulness and limitations of these models. It is imperative to note that these models should be used only to estimate resource requirements. They do not indicate how supply chains need to be designed and operated to meet these needs. Thus, they provide the basis for understanding the scope of the problems facing decision-makers but do not indicate how to address them.

## METHODS

Our focus is on models that help with both caseload projection and hospital capacity management. We conducted our review according to the Preferred Reporting Items for Systematic Reviews and Meta-Analyses (PRISMA) guidelines.^10^

### Search Strategy

We conducted our study from May 15, 2020, to July 15, 2020. The date of the last performed search was July 9, 2020. We completed database searches using PubMed (National Library of Medicine), EMBASE (Elsevier), the Institute for Scientific Information (ISI) Web of Science (Thomson Reuters), and Google Scholar. We used regular Google searches to identify additional models that were created by researchers that are publicly available on university websites. The search key words included “COVID,” “hospital,” “surge,” “estimate,” “predict,” “bed,” “caseload,” “capacity,” “tool,” and “model.” We used the search key words together with OR and AND as follows. To search the academic literature in PubMed and EMBASE, we entered: “COVID” AND (“tool” OR “model”) AND (“hospital” OR “surge”) AND (“estimate” OR “estimating” OR “predict” OR “predicting” OR “bed” OR “caseload” OR “capacity”). In ISI Web of Science, we entered: “COVID” AND (“tool” OR “model”) AND (“hospital” OR “surge” OR “estimate” OR “estimating” OR “predict” OR “predicting” OR “bed” OR “caseload” OR “capacity”). In Google Scholar, we entered: “COVID” AND “hospital” AND “surge” AND (“estimate” OR “estimating” OR “predict” OR “predicting”) AND (“bed” OR “caseload”) AND “capacity” AND (“tool” OR “model”). Finally, using the regular Google search engine, we entered: “COVID” AND “hospital” AND “surge” AND (“bed” OR “caseload”) AND “capacity” AND “tool” AND “model.”

### Inclusion Criteria

The first inclusion criterion was to ensure that the article described a computer model or tool. As a second criterion, the article needed to describe a model or tool for COVID-19. The third inclusion criterion was that the article must have investigated surge capacity management, including hospital occupancy. Fourth, we ensured that the model input parameters included the possibility to define a population served by a single hospital. The fifth criterion was that the model had to include at least 1 parameter pertaining to hospital LOS. Sixth, we ensured that the model considered ventilator capacity.

### Exclusion Criteria

We limited our search to English language articles. Second, we excluded models that focused on forecasting cases or the epidemic curve without hospital parameters. Third, we excluded models that focused primarily on the impact of non-pharmaceutical interventions on potential epidemic curves. Finally, we also excluded models that focused on hospital resources without consideration of COVID-19 caseloads or hospital LOS.

## RESULTS

**FIGURE 1:**
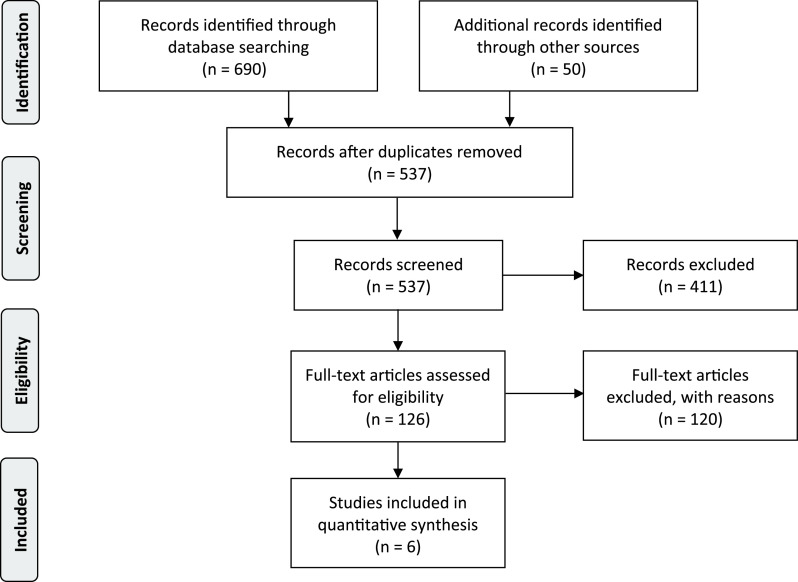
PRISMA Flow Diagram for the Systematic Review.

Our search returned a total of 690 articles. This number includes all records returned from PubMed, EMBASE, ISI Web of Science, and Google Scholar plus additional records identified from the first 50 results returned from the regular Google search query. Figure 1 provides a flow diagram to illustrate the search and selection process according to PRISMA guidelines.^10^ After eliminating duplicates, 537 articles remained. For PubMed, EMBASE, ISI Web of Science, and Google Scholar, we screened titles for possible surge capacity planning models. We then read abstracts and full-text articles of the remaining 126 articles. For regular Google search results, we visited each link to determine whether the link referred to a hospital surge capacity planning model. After considering all inclusion and exclusion criteria, we selected 6 models for a detailed review. The other articles did not meet the inclusion criteria or the exclusion criteria because (i) 19 articles did not describe a computer model, (ii) 25 articles used a population from a larger region such as a nation or state without the option for a hospital level analysis, and (iii) 22 articles focused on the impact of non-pharmaceutical interventions on potential epidemic curves. In addition, 17 papers reported models that did not include an epidemic curve, and 37 had models that did not have at least 1 parameter pertaining to hospital LOS.

Academic researchers created 5 of the models included in the detailed review and the US Centers for Disease Control and Prevention (CDC) created the sixth model. All models included in this review are available at no cost, either through an online user interface or as a spreadsheet tool available for downloading. A brief description of each model is provided along with the model’s input parameters, key features, and output. A comparison of the 6 models follows in the *Discussion* section.

### Cornell COVID-19 Caseload Calculator With Capacity and Ventilators (C5V)

This scalable interactive tool was designed to estimate the number of COVID-19 caseloads and project the critical resources needed to treat said cases for any user designed scenario. For the epidemic curve, the tool provides the option to model 1 wave, 2 waves, or use an empirical distribution supplied directly by the user. With a single wave, COVID-19 hospital admissions are assumed to be distributed according to a gamma distribution with a median of 30–90 days and dispersion parameter ranging from a relatively peaked to a relatively flat-looking arrival curve. The optional second wave can be distributed according to another gamma distribution with a median day of up to 1 year, and the dispersion parameter can be different from the first wave. Given the scenario, the hospital system projections are broken down into medical-surgical and ICU beds and ventilators while considering a variety of outbreak characteristics described in Supplementary Table 1, such as population and time period. There are 3 versions of this model available: online, Microsoft Excel (Microsoft, Redmond, WA) spreadsheet, and a desktop Python application.

We provide the C5V model parameters and output in Supplementary Table 1. Access to the online version of C5V^11^ is available by clicking the sign up link and entering your email and password. The web site has links to a video demonstration and to other versions of the C5V. A recent working paper provides additional information^12^.

### COVID-19 Acute and Intensive Care Resource Tool (CAIC-RT)

This online tool provides an estimate of the maximum manageable daily number of incident COVID-19 cases that a health care system could serve based on an age-stratified case distribution and severity, as well as available medical resources, such as the number of available acute and critical care beds. Created at the University of Toronto, Ontario, Canada, in collaboration with University-affiliated health networks, the authors provided versions in 3 different languages: English, French, and Spanish. Supplementary Table 2 provides the model parameters and output for the CAIC-RT. The source code and a video demonstration are available online,^13^ and a research paper provides additional information.^14^

### COVID-19 Hospital Impact Model for Epidemics (CHIME)

The COVID-19 Hospital Impact Model for Epidemics (CHIME) is an online tool developed by the Predictive Healthcare Team at the University of Pennsylvania in Philadelphia. It offers users the ability to visualize forecasts for several outcomes of the COVID-19 outbreak – for example, cumulative number of hospitalizations, number of new daily hospitalizations, and cumulative number of susceptible individuals in a population. Hospital administrators, personnel, and public health officials can use it to predict caseload and epidemic curves, to adjust medical resources accordingly, and, overall, to enable a data-driven resource requirements plan for responding to the outbreak. CHIME also offers an optional spreadsheet-based PPE tool.

CHIME uses a discrete-time susceptible, infected, removed (SIR) model. The parameters are estimated from “other locations … based on logical reasoning, and best guesses from the American Hospital Association.”^15^ While some model parameters cannot be changed directly, CHIME has parameters that can be changed. We provide those parameters and the output in Supplementary Table 3. A video demonstration is available on the CHIME website,^15^ and a research paper provides additional information.^16^

### COVID-19 ICU and Floor Projection

Stanford University’s COVID-19 ICU and Floor Projection Model is an online model designed to facilitate hospital planning by estimating bed demand for COVID-19 patients. The model estimates the daily number of COVID-19-related medical resources required, such as intensive care beds, acute care beds, and ventilators necessary to balance the hospitalization-required patient population and hospital capacity.

The model is available on the Systems Utilization Research for (SURF) Stanford Medicine website.^17^ We provide the model parameters and output in Supplementary Table 4. A recent working paper provides additional information.^18^

### COVID-19Surge

COVID-19Surge is a spreadsheet-based tool created by the US CDC that can be used to estimate the surge in demand for hospital resources during the COVID-19 pandemic. Users can estimate the number of COVID-19 patients with different needs, such as hospitalization, ventilators, and ICU. At the same time, users can input the current number of patients and available medical resources to assess which community mitigation strategy is more appropriate. This allows a comparison of the predicted value with the existing resources of the hospital to make a reasonable allocation.

Model parameters and output are provided in Supplementary Table 5. The model can be downloaded from the CDC website,^19^ where additional documentation is available.

### Surge Capacity Bed Management Tools

This tool is a spreadsheet-based model that helps project up to 30 days in advance for hospital bed demand and occupancy (census), ICU beds, critical equipment, and PPE consumption, also known as the *burn rate*. This model uses both deterministic and random options to calculate predictions and also has an accuracy tracker to ensure proper inputs and outputs. By providing inputs such as admission rates and LOS for medical, ICU, and ventilated patients, the model can be used to help address capacity concerns, supply consumption concerns, as well as operational decisions amid the COVID-19 pandemic.

We provide the model parameters and output for the Bed Demand Tool in Supplementary Table 6. Optional functionality for PPE and staff planning is excluded from Supplementary Table 6. The entire spreadsheet model is available freely to any health system and can be downloaded from Northeastern University’s Healthcare Systems Engineering Institute website.^20^ A recent working paper provides additional information.^21^

## DISCUSSION

Public health officials and hospital leaders worldwide continue to face unprecedented challenges due to the COVID-19 pandemic. Uncertainty existed and still exists about the disease’s spread over time. This uncertainty makes resource planning exceptionally difficult. Models used to estimate resource needs are based on assumptions of how a pandemic occurs over time. In particular, parameter values used in a specific scenario represent a user’s estimate of a possible way that a pandemic and its response might arise. For example, the age-stratified CDC modeling parameters^22^ changed from their earliest iterations in mid-February 2020 to the later version in April 2020; the “true” values for many parameters relating to COVID-19 hospitalization may still be quite different from the CDC estimates. A recent editorial compared Penn’s CHIME model with University of Toronto’s CAIC-RT model. Comments include: “As with hurricane-tracking models, they make varying projections; yet in the face of uncertainty, they provide useful real-time forecasts to prepare for the pandemic, as evidenced by their broad use.”^23^ It can be misleading to run a single scenario and report it as a forecast of what is going to happen. Instead, multiple scenarios should be run with a variety of parameter values. The models included in this review would be used as intended only if multiple scenarios are examined.

### Model Comparison

Table 1 provides a summary of the similarities and differences of the 6-hospital surge capacity planning models we reviewed. Three of the models have online interfaces only, 2 have spreadsheet interfaces only, and Cornell’s C5V has all 3 versions: an online version, a spreadsheet version, and a desktop version. The University of Toronto’s CAIC-RT model is available in English, French, and Spanish, whereas the other 5 models are offered in English only. For most models, the planning horizon is limited to 30 days. Exceptions are versions of Cornell’s C5V (the spreadsheet version of which covers pre- and post-peak periods up to 180 days, and the online version of which can model up to 360 days) and the spreadsheet version of the CDC COVID-19Surge that supports a 1-year planning horizon. A longer planning horizon is particularly helpful for modeling an outbreak with multiple waves. Most of the tools provide the option to model a single wave, whereas the online version of Cornell’s C5V is the only tool that provides the option to model a second wave.

**TABLE 1:** Comparing COVID-19 Hospital Surge Capacity Planning Models

MODEL NAME	AUTHOR AFFILIATION	VERSION	PLANNING HORIZON	POPULATION AGE STRATIFICATION	HOSPITALIZATION, ICU PROPORTION	EPIDEMIC CURVE		LENGTH OF STAY (LOS)	OUTPUT
						FIRST WAVE	SECOND WAVE		
Cornell COVID-19 Caseload Calculator with Capacity and Ventilators (C5V)	Weill Cornell Medicine, Singapore University of Technology and Design, San Jose State University, Cornell Engineering	Online	180 to 360 days	7 age ranges: 0-19, 20-44, 45-54, 55-64, 65-74, 75-84, 85+ or 5 age ranges: 0-4, 5-17, 18-49, 50-64, 65+	Included with age stratification	Gamma distribution or empirical distribution	Gamma distribution or empirical distribution	Min and max LOS for adult medical-surgical, pediatric medical-surgical, adult critical care, pediatric critical care; includes fatality ratios with LOS adjustments, different LOS for critical care patients without ventilator; uniform distribution with min, max to determine LOS for each type	On screen graphs, option to download.xlsx file with all data and all graphs included
		Spreadsheet	180 days	5 age ranges: 0-4, 5-17, 18-49, 50-64, 65+		Gamma distribution	None		Output worksheet with all data and all graphs included
		Desktop (Python)							On screen graphs, output can be copied to.xlsx file using “Print” button
COVID-19 Acute and Intensive Care Resource Tool (CAIC-RT)	Dalla Lana School of Public Health, Department of Medicine, University of Toronto	Online	N/A	7 age ranges: 0-19, 20-44, 45-54, 55-64, 65-74, 75-84, 85+	Included with age stratification	N/A	N/A	Average LOS in hospital, in ICU, on ventilator	Single graph with max number of hospital, ICU beds, ventilators with option to download.pdf file report with data and graph
COVID-19 Hospital Impact Model for Epidemics (CHIME)	Perelman School of Medicine, University of Pennsylvania	Online	30 days	None	Included	Discrete-time SIR model	None	Average LOS in hospital, in ICU, on ventilator	On screen graphs, option to download separate.csv file with data used in each graph (graph excluded)
COVID-19 ICU and Floor Projection	Stanford Medicine, Stanford Graduate School of Business, Management Science and Engineering, Stanford University	Online	34 days (default)	None	Included	Exponential distribution	None	Average LOS for different patient pathways: Floor only, Floor to ICU to Floor, Floor to ICU, ICU to Floor, ICU only	On screen graphs and on screen tabular data provided
COVID-19Surge	Centers for Disease Control and Prevention (CDC)	Spreadsheet	365 days	5 age ranges: 0-4, 5-17, 18-49, 50-64, 65+	Included with age stratification	Exponential or uniform distribution	None	Average LOS in hospital, in ICU, on ventilator	Spreadsheet output with tabular data and graphs
Surge Capacity Bed Management Tools	Healthcare Systems Engineering Institute, Northeastern University	Spreadsheet	30 days	None	Included	Piece-wise exponential or empirical distribution	None	Average LOS for different patient pathways: medical-surgical only, medical-surgical to ICU with ventilator, medical-surgical to ICU without ventilator, ICU without ventilator to medical-surgical, ICU with ventilator only, ICU without ventilator only	Spreadsheet output with tabular data and graphs

Studies show that the odds of death from COVID-19 increase with age with different proportions reported in Wuhan, China,^24^ Northern Italy,^25^ and NYC.^26^ Furthermore, an NYC study of 5700 patients hospitalized with COVID-19 reported that LOS and ventilator needs differ by age group.^26^ Therefore, another important distinguishing feature is age-stratification, usually based on CDC modeling parameters.^22^ Naturally, the CDC COVID-19Surge supports age-stratification but, surprisingly, only for the 5 age ranges originally released, not the newer division into 7 age ranges. The online version of the University of Toronto’s CAIC-RT model supports the newer 7-strata age distribution, the spreadsheet and desktop versions of C5V support the older 5 age ranges, and the online version of C5V provides the option to use either 7 age ranges or 5 age ranges. The other 3 models do not include age-stratification. Hence, they use the same hospitalization and ICU proportions in their models, regardless of a patient’s age. For the epidemic curve, some modelers adopted a mechanistic approach. In some cases, this requires inputting a parameter, called the *doubling time*: the number of days it takes for cases to double. With the doubling time as a parameter, mechanistic models assume that the number of cases will grow exponentially. For example, Penn Medicine’s CHIME model uses a doubling time that is configurable and other parameters are based on estimates and the shape of curves observed at the beginning of the COVID-19 outbreak in China and Italy. The University of Toronto’s CAIC-RT model takes a different approach. Instead of the epidemic curve, the model focuses on identifying the maximum number of patients that the hospital can handle in a surge situation.

In practice, there are different patient pathways for COVID-19 patients. For example, a patient could be admitted to a medicine ward and later be transferred to the ICU and need a ventilator. However, most models have the simplifying assumption that each patient’s entire LOS will only be in a medical-surgical ward, in the ICU with a ventilator, or in the ICU without a ventilator. Exceptions are SURF Stanford Medicine and Northeastern University’s models that consider different patient pathways and provide the option to set different LOS for each pathway.

Most models further simplify the complexity of hospital patient flow by using average LOS. However, the LOS used in Cornell’s C5V is set randomly according to a uniform distribution with minimum and maximum parameters set by the user. The C5V also includes fatality ratios with LOS adjustments for survivors versus those who die, and also accommodates different LOS for critical care patients who do or do not require a ventilator.

On screen output with tabular data and graphs is available for all 6 models. Considering that many users may wish to perform additional analyses or generate reports, the majority of the tools include options to download output to a spreadsheet. The download option for the University of Toronto’s CAIC-RT model is a.pdf file report, whereas the SURF Stanford Medicine model provides tabular data and graphs on screen only. The models from the CDC, Cornell University, and Northeastern University provide the user with the option to download a spreadsheet with output that includes both tabular data and graphs, whereas Penn’s CHIME model provides the option to download tabular data without graphs.

### Strengths and Limitations

The main goal of this review was to identify models that can project both COVID-19 caseload and surge capacity requirements over time for hospital level analysis with parameters including LOS, occupancy, and ventilator capacity. We provided detailed documentation with the input parameters, key features, and explained the output that can be produced from each model. We also provided a comparison table to highlight the similarities and differences of the models that are available to assist in this planning process. The details provided in this review may help physicians, hospital administrators, emergency response personnel, and governmental agencies evaluate models for preparing scenario-based plans for responding to the COVID-19 or similar type of outbreak.

There are other existing models that are useful but did not meet the inclusion criteria or the exclusion criteria for this study. For example, the University of Washington’s Institute for Health Metrics and Evaluation (IHME) model^27^ is widely used for COVID-19 projections. The IHME model supports analysis at the country or state level, whereas the models reviewed in this paper can specify a population served by a hospital, hospital network, region, state, or nation. The IHME model is also different in that it does not have the option to enter all the user-defined parameters that the models in this review include – especially relating to hospital LOS and capacity.

Without a vaccine for COVID-19, communities around the world are sheltered in place and engaged in physical distancing to try to reduce the spread of COVID-19. Naturally, with the emphasis on distancing and other non-pharmaceutical interventions (NPIs), there are also many models emerging that focus on NPIs. Some of the models included in this review include NPIs. However, there are other models that focus more on NPIs than the models included in this review. For example, the COVID-19 International Modelling (CoMo) Consortium model considers many different NPIs, including handwashing, working at home, school closures, international travel ban, vaccination, shielding the elderly, and self-isolation.^28^ It also models health care capacity but does not yet have a complete description in a working paper.

The models included in this systematic review can help predict and prevent health system capacity constraints by estimating hospital bed and ventilator requirements before they reach a crisis point. Due to shortages for critical health care resources, including PPE, some of the models included in this review also include a PPE calculator. These and future models may buttress the global health care supply chain’s preparedness for challenges caused by the first wave and potentially subsequent waves of COVID-19.

## CONCLUSION

The COVID-19 pandemic continues to create extraordinary challenges for hospital leaders. In this systematic review, we identified and reviewed surge capacity planning models that handle both caseload projection and hospital capacity management for this novel pandemic disease. These models have key differences: some can be used for a longer planning horizon, some have age-stratified parameters, and some incorporate different patient pathways and more detailed patient flow. An enhanced understanding of model similarities and differences may help physicians, hospital administrators, emergency response personnel, and public health agencies determine which existing models are appropriate for their use. These models help users quantify resource requirements over time for a particular set of scenarios, providing a quantitative way to describe complex health system capacity constraints under COVID-19. The crucial problem now facing health systems worldwide is to determine how to construct and operate the complex supply chain needed to create the required resources.

## Supplementary material

For supplementary material accompanying this paper visit http://dx.doi.org/10.1017/dmp.2020.332. click here to view supplementary material
