# Supplementary material for: COVID-19 Models for Hospital Surge Capacity Planning: A Systematic Review
Source: Disaster Med Public Health Prep. 2020 Sep 10:1–8. doi: 10.1017/dmp.2020.332 (PMC7643009; doi:10.1017/dmp.2020.332)
Supplement: Supplementary file 1 [file S1935789320003328sup001.docx]

ONLINE SUPPLEMENT

COVID-19 Models for Hospital Surge Capacity Planning: A Systematic Review

Michael G. Klein, Ph.D.*, Carolynn J. Cheng, B.S., Evonne Lii, B.S., Keying Mao, Hamza Mesbahi, B.S., Tianjie Zhu, B.S.
Department of Marketing & Business Analytics, San Jose State University

John A. Muckstadt, Ph.D.
School of Operations Research & Information Engineering, and Cornell Institute for Disease and Disaster Preparedness, Cornell University

Nathaniel Hupert, MD, MPH

Departments of Population Health Sciences and of Medicine, Weill Cornell Medicine, and Cornell Institute for Disease and Disaster Preparedness, Cornell University

*corresponding author address: Department of Marketing & Business Analytics, San Jose State University, One Washington Square, San Jose, CA 95192-0069, phone: 408-924-3520, fax: 408-924-3534, email: [michael.klein@sjsu.edu](mailto:michael.klein@sjsu.edu)

Table 1 - Cornell COVID Caseload Calculator with Capacity and Ventilators (C5V)

| **REQUIRED MODEL INPUT** | **NOTES** |
| --- | --- |
| **Step 1: Population**   - Enter the age-stratified population for a single hospital, hospital network, region, state, or nation - Select either a 5 or 7 age range option based on CDC modeling parameters [13] - Input the Total Area Population for each Age Range | - *Online Version:* You have the ability to save scenarios and populations under a customizable name - Default Scenarios (ex: DEFAULT_S1, DEFAULT_S2) and Populations (ex: NYC, NYC_2) are provided as an example of how to use the tool |
| **Step 2: Attack Rate, Percent Symptomatic**   - Enter your assumed overall attack rate (infection rate) and assumed % of symptomatic people | - Attack rate is the % of population that will eventually become infected, both symptomatic and asymptomatic |
| **Step 3: Hospitalization and ICU Proportions**   - Enter the age-stratified assumed case hospitalization ratio (CHR) and assumed critical care hospitalization fraction (CCHF) for both Mild and Severe Attack Scenarios per Age Range | - You have the option to save CHR and CCHF parameters to your account - Default parameters are based on CDC modeling parameters [13] |
| **Step 4: Epidemic Curve**   - For *Online Version:* Select either a “gamma” or “empirical” epidemic curve probability density function (PDF) - For *other versions*, only “gamma” PDF is available - Enter the date for the Start of Curve - For *Online Version:* Select a duration (total number of days) - For *other versions*, the planning horizon is 180 days - Choose the day for the maximum cases - Choose the shape of the epidemic curve (from Broad to Extremely Peaked) | For *Online Version:*   - If “empirical” PDF is selected, select the duration and provide the empirical distribution for the selected number of days (The values should sum to 1). - If a *Second Wave* is desired, select “Yes” in the drop-down list and provide the following:   - First Wave proportion   - The day of maximum cases for the Second Wave   - The shape of the Second Wave of the epidemic curve (ex: Broad to Extremely Peaked) |
| **Step 5: LOS, fatality ratios** **with adjustments**  For regular and critical care COVID-19 patients per Adult and Pediatric care Ward   - Input the Estimated LOS - Provide the minimum and maximum days for LOS - Provide the Fatality Ratio for each category - Provide the LOS adjustment for each category   Provide the above information for Critical Care Patients without Ventilators for Adults and Pediatrics with Mild and Severe cases | - LOS inputs for regular and critical care COVID-19 patients are assumed common for both mild and severe populations |
| **Step 6: Bed and Ventilator Capacity**   - Enter the Quantity Available for the following resources:   - Medical/Surgical Ward Beds   - Critical Care Beds   - Critical Care Ventilators |  |
| **OUTPUT** | **NOTES** |
| A graph with the key scenarios will display with the following chart types:   - Expected Number of Cases (default) - Patient Daily Census by Location (Unlimited Capacity) - Occupancy by Bed Type (Limited Capacity) - Cumulative Overflow - ICU Admissions, Access Denials and Ventilator Shortage Deaths | - For each chart type, you can select between Mild and Severe scenarios - For Expected Number of Cases and Patient Daily Census, you can specify whether you want to display Adult, Pediatric, or All data. - Model Output can be downloaded with all data and graphs as a MS Excel file for further analysis - For *spreadsheet version*, view the various key scenarios by selecting the OUTPUT PAGE worksheet |

Table 2 - COVID-19 Acute and Intensive Care Resource Tool (CAIC-RT), University of Toronto

| **REQUIRED MODEL INPUT** | **NOTES** |
| --- | --- |
| **Step 1: Review Age-stratified Case Distribution and Severity based on CDC modeling parameters [13]**   - Age Groups - Case Distribution (%) - Require Acute Care (%) - Require Critical Care (%) | - Upload a CSV or Excel file with the four columns shown on the left - Percent of critical care patients requiring mechanical ventilation (0-100%) |
| **Step 2: Tune Expected Resource Utilization**   - Mean days in acute care - Mean days in critical care - Mean days on a mechanical ventilator |  |
| **Step 3: Enter Resource Availability**   - Total Number of acute care beds for COVID-19 patients - Total Number of critical care beds for COVID-19 patients - Total Number of mechanical ventilators for COVID-19 patients | For each input, you can also customize the % of resource available or currently being used by COVID-19 cases along with surge capacity  Input parameters for calculation (*Formula: Total number * percent + surge capacity*)   - Total number of acute/ critical care beds/ mechanical ventilators - Percent of acute/ critical care beds/ mechanical ventilators available or currently being used by COVID-19 cases - acute/critical care beds/ mechanical ventilators surge capacity for COVID-19 cases (number of beds) |
| **OUTPUT** | **NOTES** |
| **Maximum Daily number of incident cases manageable by healthcare system**   - Thresholds for the max daily number of incidents that can occur without causing a resource deficit - Bar graph showing: Acute Care Beds, Critical Care Beds, and Mechanical Ventilators (Cases/Day) hospital capacity | - You can change the bar chart color in the settings - Interpretation of results provided for Acute Care, Critical Care and Mechanical Ventilators |
| **Generate a PDF report of the results and also bookmark custom inputs into a website link** |  |

Table 3 - COVID-19 Hospital Impact Model for Epidemics (CHIME), University of Pennsylvania

| **REQUIRED MODEL INPUT** | **NOTES** |
| --- | --- |
| **Hospital parameters:**   - Region population (default = 3,600,000) - Hospital market share % (default = 15) - Currently hospitalized COVID-19 patients (default = 69) |  |
| **Spread and Contact parameters:**  If “I know the date of the first hospitalized case” is selected, then only that date is required; if not, then state a “doubling time in days.” By default, “I know the date of the first hospitalized case” is not selected, and doubling time = 5 | Optional social distancing feature:  1) Input implementation date of policy  2) Input % reduction in social contact while policy is in effect |
| **Severity parameters:**   - Hospitalization % (of total infections) (default = 2.5) - ICU % (of total infections) (default = .75) - Ventilated % (of total infections) (default = .5) - Infectious days (default = 10) - Average hospital length of stay (in days) (default = 7) - Average days in ICU (default = 9) - Average days on ventilator (default = 10) |  |
| **Display parameters:**   - Number of days to project (default = 30, max = 30) - Current date (default = today) | Optional:   - “Set the Y-axis on graphs to a static value.” If selected, then state static value (default = 500) - “Use logarithmic scale on charts instead of linear scale” |
| **OUTPUT** | **NOTES** |
| **General Text**   - Number of currently infected individuals - R_o_ (Basic reproductive number) - the expected number of cases directly generated by one case in a population where all individuals are susceptible to infection. - Daily growth rate % (Infected people) | The general output is based on the initial input (by default without social distancing measures). |
| **Mitigation**   - Doubling time - R_t_ (Effective reproductive number) - the average number of secondary cases per infectious case in a population made up of both susceptible and non-susceptible hosts. - Daily growth rate % (Infected people) | If social distancing feature is selected, then doubling time, R_t_, and daily growth rate change. |
| **New Admissions (Interactive line chart)**  Projected number of daily COVID-19 admissions   - Hospitalized Admissions - ICU Admissions - Ventilated Admissions | Can download table as a .csv file |
| **Admitted Patients (Census) (Interactive line chart)**  Projected census of COVID-19 patients, accounting for arrivals and discharges   - Hospitalized Census - ICU Census - Ventilated Census | Can download table as a .csv file |
| **Susceptible, Infected, and Recovered (Interactive line chart)**  The number of susceptible, infected, and recovered individuals at any given moment   - Infected - Recovered - Susceptible | Can download table as a .csv file |

Table 4 - Covid-19 ICU and Floor Projection, Stanford University

| **REQUIRED MODEL INPUT** | **NOTES** |  |
| --- | --- | --- |
| **General Census Data_1 (Template 1)**   - ICU COVID Census - ICU non-COVID Census - Floor COVID Census - General Medicine Floor non-COVID Census   **General Census Data_2 (Template 2)**   - Cumulative Number of COVID Admits | - The inputs are separated in two .csv files and have to be uploaded with a specific format. - The range of the input is from March 13 to Apr 15 (34 days). - The inputs consider both COVID patients and non- COVID patients. - No default number - Need to fill in the numbers before uploading the spreadsheet | |
| **General Parameters**   - Number of Days to Project - Doubling Time for New COVID Admits - Capacity of ICU - Capacity of General Medicine Floor - Ventilator Capacity - Fraction of Non-COVID ICU Patients in need of Ventilators （%） |  | |
| **Hospital Starting Status Parameters**   - Cumulative Number of Admitted COVID Patients by Day 0 - ICU Census of COVID patients at Day 0 (note if census data was inputted, these parameters are irrelevant) - General Medicine Floor Census of COVID patients at Day 0 (note if census data was inputted, these parameters are irrelevant) - Mean Daily ICU Census of non-COVID patients - Mean Daily General Medicine Floor Census of non-COVID patients |  | |
| **COVID Patient Population Parameters**   - Floor Only:% - Floor to ICU to Floor:% - Floor to ICU:% - ICU to Floor:% - ICU Only:% | - Input the % breakdown of COVID patients who go through the five different paths. These must sum to 1. - Use range gauges to input data | |
| **LOS Parameters**   - Floor Only - Floor to ICU to Floor - Floor to ICU - ICU to Floor - ICU Only | - Input the **average** LOS (in days) in each unit for various COVID patient cohorts. Note the second “Floor” column is for patients who are coming from the ICU. | |
| **OUTPUT** | **NOTES** |  |
| Tabular on screen output (Template 1)   - Date (from Mar 13 to Apr 15) - ICU COVID Census - ICU non-COVID Census - Floor COVID Census - Floor non-COVID Census   Tabular on screen output (Template 2)   - Date (from Mar 13 to Apr 15) - Total COVID Admit | - The on screen output has the same format as the given templates. - The results are separated into COVID Census and non-COVID Census | |
| **Graphical Representation**   - Line chart regarding the projected ICU Census   - X-axis: Date   - Y-axis: Number of patients - Line chart regarding the projected general medicine IP floor Census   - X-axis: Date   - Y-axis: Number of patients |  | |
| **Tabular Representation**   - A table showing the ICU Census and Floor Census   - Day   - ICU COVID Census   - ICU Non-COVID Census   - Total ICU Census   - Floor COVID Census   - Floor Non-COVID Census   - Total Floor Census | - You can choose to show 10/25/50/100 entries - There is a search engine - Ventilator is not included | |
| **Sensitivity Analysis**   - Doubling time   - A table outlines the number of days until demand exceeds the ICU capacity, Floor capacity from Mar 13. - Impact of a one day LOS decrease/increase on the days until demand exceeds floor capacity   - A table outlines the number of days until demand exceeds the Floor capacity starting from Mar 13. - Impact of a one day LOS decrease/increase on the days until demand exceeds ICU capacity   - A table outlines the number of days until demand exceeds the ICU capacity starting from Mar 13. - Impact of a one day LOS decrease/increase on the days until demand exceeds ventilator capacity   - A table outlines the number of days until demand exceeds the ventilator capacity starting from Mar 13. | - The impact part is divided into 3 parts for hospital capacity planning: 1) until demand exceeds floor, 2) ICU, 3) ventilator capacity. | |
| **Analysis of Cumulative COVID Admissions**   - Based on the input parameters, it shows a table consisting of the number of data point used, fitted doubling time, and the fitted cumulative number of admissions per day - Line chart showing the cumulative COVID Admissions and fitted values |  | |

Table 5 – COVID-19Surge, Centers for Disease Control and Prevention (CDC)

| **REQUIRED MODEL INPUT** | **NOTES** |  |
| --- | --- | --- |
| **Part A: Hospital Resource**   - Step 1: Enter the population: (default: 1000000) - Step 2: Enter basic hospital resources - Total staffed, unoccupied non-ICU beds - Total staffed, unoccupied ICU beds - Total number of ventilators available | The user can select a population type, such as a local population or any other relevant population. | |
| **Part B: Hospital Stays for COVID-19 Patients**   - Step 1: Enter information about non-ICU COVID-19 patients - **Average** % of admitted COVID-19 cases who will be admitted for hospital care - **Average** length of non-ICU hospital stay - Step 2: Enter information about ICU COVID-19 patients - **Average** % of admitted COVID-19 cases who will require ICU care - **Average** duration in ICU (if no ventilator) - Step 3: Enter information about ventilator use - **Average** % of COVID-19 cases in ICU that will need ventilators - **Average** duration in ICU (if ventilator required) - **Average** downtime per ventilator (decontamination/relocation) | Default values for average hospital LOS:   - Non-ICU: 8 days - ICU, no ventilator: 10 days - ICU, with ventilator: 16 days | |
| **Part C: Community Outbreak Details**   - Step 1: today’s date (or date for start of analysis) - Step 2: Information about case counts in your jurisdiction - Total cases to date analysis starts - Cases in the **14 days** before analysis starts - Step 3: Estimates about the effectiveness of community-level interventions - Data intervention began/begins - Planned duration of intervention (days) - Effectiveness of interventions : % of decrease in # of new infections per case | - Step 3: There are four options for intervention: No Intervention, Intervention 1: Low Intensity, Intervention 2: Medium Intensity, Intervention 3: High Intensity - Optional part: Users can calculate age-weighted population values. | |
| **Part D: Epidemiological Parameters (Optional/Advanced)**   - Information about disease stages - Infected (Not Contagious) Period - Contagious Period - Convalescent Period (non-hospitalized) - Total duration of infection/illness - Enter information about how quickly the disease spreads - New Infections per Case (low estimate) - New Infections per Case (high estimate) | Default is exponential distribution, uniform distribution can also be selected | |
| **Part E: Calculate Age-Weightings (Optional/Advanced)**   - Step 1: Demographic information for your jurisdiction - % of Population aged 0-4 years - % of Population aged 5-17 years - % of Population aged 18-49 years - % of Population aged 50-64 years - % of Population aged 65+ years - Step 2: Age-specific risk for parameter of interest for each age group - Probability (0-4 years) - Probability (5-17 years) - Probability (18-49 years) - Probability (50-64 years) - Probability (65+ years) |  | |
| **OUTPUT** | **NOTES** |  |
| **Main Summary**   - **Graphs -** three graphs that illustrate differences in bed occupancy and ventilator use and three social distancing scenarios. | - Graphs with five types: No Intervention; Intervention 1: Low Intensity; Intervention 2: Medium Intensity; Intervention 3: high Intensity; Unoccupied, staffed capacity. - There are two rates of transmission that can be chosen : lower (R_0_ = 2.0) or higher (R_0_ = 2.6) - a red-dotted line is available in these graphs, which represents the capacity for non-ICU beds, ICU beds, and ventilators (entered in part A) | |
| **Demand Summaries**   - **Graphs -** four graphs that illustrate each of the intervention scenarios showing estimated demand for hospital resources (beds, ICU beds, ventilators) | - These estimates of demand can be compared with hospital capacity entered in part A - Analysis based on higher and lower estimates of disease transmission. - Users can click “More Details” to get more information about each intervention scenario | |
| **Detailed Results**   - **Graphs -** graph illustrates ICU demand compared to hospital capacity (entered in part A) - **Tabular information** - Total ICU Admissions - Peak ICU Admissions - ICU Occupancy | - Based on higher and lower estimates of transmission | |

Table 6 - Surge Capacity Bed Management Tools, Northeastern University

| **REQUIRED MODEL INPUT** | **NOTES** |
| --- | --- |
| **Step 1: COVID-19 Rates and Admission Trends**   - Enter Today’s Date - Select the planning period - Select either “manual” or “doubling trend” for suspected cases trend - Enter the number of days it takes for cases to double the number of suspected cases - Enter the recent number of suspected cases for the day before “Today’s Date” - Enter the percentage of suspected cases to admit   For Non-COVID Patients   - Select either “manual” or “daily” for the Non-COVID trend - Enter the Number of daily non-COVID admits - Enter the percentage of non-COVID cases to defer   Calculation Mode   - Select either a “deterministic” or “random” calculation mode - Enter the percent variation for all LOS | - Doubling rate is assumed to be piecewise exponential curve - For number of daily non-COVID admits, all inputs are assumed to be deterministic |
| **Step 2: Current Patient Census**   - Enter the number of COVID and Non-COVID *MedSurg* Patients starting on Day 1 - Enter the number of COVID and Non-COVID *ICU* Patients starting on Day 1 - Enter the number of COVID and Non-COVID *ICU ventilated* Patients starting on Day 1 | - All inputs are for Day 1 |
| **Step 3: COVID Patients LOS & Location Percentages**  For COVID PATIENTS:   - Enter the average length of stay in days for COVID and Non-COVID patients in: MedSurg, ICU, and ICU ventilated - Enter the Percentages of new patients admitted to MedSurg, ICU beds without ventilators, and ICU ventilated - Enter the percent of ICU patients stepping down to MedSurg and their LOS in MedSurg - Enter the percent of ventilated patients staying in ICU post-extubation and their LOS in ICU - Enter the percent of MedSurg patients escalating to ICU | - The three Percentages of admission should add to 1 - % of ICU patients in ICU or ICU ventilated that are NOT transferred to MedSurg or extubated are implied deaths or transfers |
| **Step 4: Regular Patients LOS & Percentages**  For REGULAR (NON-COVID) PATIENTS:   - Enter the average length of stay in days for COVID and Non-COVID patients in: MedSurg, ICU, and ICU ventilated - Enter the Percentages of new patients admitted to MedSurg, ICU beds without ventilators, and ICU ventilated - Enter the percent of ICU patients stepping down to MedSurg and their LOS in MedSurg - Enter the percent of ventilated patients staying in ICU post-extubation and their LOS in ICU - Enter the percent of MedSurg patients escalating to ICU |  |
| **Step 5: Health System Capacities**   - Enter the number of COVID and Non-COVID beds available in: MedSurg and ICU - Enter the number of ventilators currently available | - Enter the number of beds regardless of whether they are currently occupied or empty - Optional: Enter the number of potential additional ventilators (Default is 0) |
| **OUTPUT** | |
| Multiple graphs will display and adjust accordingly to the inputs provided.  **For COVID Patients:**   - A graph will display the number of Occupied COVID Patient Bed Demand   - Legend available for: MedSurg, ICU total, and ICU ventilate along with the bed capacities for each (dotted lines) - A graph will display the number of New COVID Patient Daily Admissions   **For Total Patients:**   - A graph will display the number of Occupied All-Patient Bed Demand   - Legend available for: MedSurg, ICU total, and ICU ventilate along with the total bed and ventilator capacities for each (dotted lines) - A graph will display the number of New All-Patient Daily Admissions   Summary Tables for Bed Demand and New Patients will offer the number of demand for beds as well as the number of new patients admitted for each day   - Bed Demand and New Patients are broken down into COVID or Non-COVID and whether they are admitted into MedSurg or ICU with Ventilators. - ICU total and the total of COVID and Non-COVID patients is provided as well | |
